# Kinetics of Iron Nitride Layer Growth during the Nitriding of AISI 1085 Non-Alloy Steel and AISI 52100 Alloy Steel

**DOI:** 10.3390/ma17184623

**Published:** 2024-09-20

**Authors:** Tadeusz Frączek, Rafał Prusak, Jerzy Michalski, Magdalena Kowalewska-Groszkowska

**Affiliations:** 1 Department of Materials Engineering, Faculty of Production Engineering and Materials Technology, Czestochowa University of Technology, 42-201 Czestochowa, Poland; tadeusz.fraczek@pcz.pl; 2 Department of Production, Faculty of Production Engineering and Materials Technology, Czestochowa University of Technology, 42-201 Czestochowa, Poland; 3 Institute of Precision Mechanics, Duchnicka 3, 01-796 Warszawa, Poland; jerzymichalski987@gmail.com; 4 Museum and Institute of Zoology of the Polish Academy of Sciences, Palmiry, ul. Wiśniowa 22, 05-152 Czosnów, Poland; mkowalewska@miiz.waw.pl

**Keywords:** gas nitriding, alloy and non-alloy steels, nitriding process kinetics

## Abstract

This paper presents a comparison of two-component ammonia-based inlet atmospheres diluted with either hydrogen (NH_3_/H_2_) or nitrogen (NH_3_/N_2_). Taking advantage of the features of inlet atmospheres diluted with nitrogen and hydrogen, four two-stage processes were designed and carried out, which were juxtaposed with two single-stage processes carried out only in an NH_3_ atmosphere. A common parameter of the processes carried out was the same value of nitrogen availability in each process stage. The gas nitriding process was carried out on ASIS 1085 non-alloy steel and ASIS 52100 alloy steel. It was found that the chemical composition of the steels studied, for the adopted nitriding process parameters, did not affect the kinetics of the growth in the mass of nitrided samples as a function of the nitriding time. However, the additions of alloying elements present in the steels studied significantly affected the nitrogen distribution between the resulting iron nitride layer and the diffusion zone in the nitrided substrate. Because of the presence of chromium in AISI 52100 steel, a larger mass of nitrogen accumulated in the nitriding zone in the solution compared with unalloyed AISI 1085 steel. As a result, with the same increase in the mass of nitrided steel, a thicker layer of iron nitrides formed on AISI 1085 steel than on AISI 52100 steel.

## 1. Introduction

Nitriding of products made of steel (and also of other materials) can be performed using various methods, in which nitrogen can exist in the following forms [1]: solid [2], liquid [3], gas [4], or plasma [5]. In industrial practice, gas nitriding is most commonly used [6]. This process is a common heat treatment method that changes the chemical composition of the surface by increasing the nitrogen content of the surface layer [7]. Nitriding results in an enhancement in corrosion resistance (due to the formation of nitride layers) and performance properties (as a consequence of increased surface hardness, wear resistance, and fatigue strength) [8,9]. The growth of a nitrided layer on iron and its alloys is described by the laws of thermodynamics and kinetics [2]. The laws of thermodynamics can be used to determine the local equilibrium between nitrogen on the steel surface and the nitriding atmosphere and the equilibrium between nitrogen in the nitride phases α, γ’, and ε and at the interphase boundaries—α/γ’ and γ’/ε, respectively. Kinetic laws include reactions at the atmosphere-metal interface and nitrogen diffusion into the substrate. Kinetic conditions can fundamentally affect the final outcome of the process [10,11]. The basic parameters characterising the nitriding atmosphere are the nitriding potential Np, degree of ammonia dissociation α, and nitrogen availability m_N2_ [12]. The first parameter—nitriding potential, which is the ratio of ammonia partial pressures to hydrogen—reflects the ability of the gas mixtures and temperature to introduce nitrogen into the sample [13] and determines the potential capacity of the nitriding atmosphere in terms of the formation of nitrogen phases α, γ’, and ε under conditions of concentration equilibrium of the nitriding atmosphere with the nitrided surface. The second parameter—the degree of dissociation—determines how much of the ammonia must decompose (dissociate) in order to provide the nitrogen in statu nascendi necessary to form the nitrided layer and reach equilibrium, defined by the nitriding potential. The third parameter, nitrogen availability, is a parameter that links the degree of ammonia dissociation with a technological parameter, i.e., the inlet atmosphere flow rate F_w_, and contains information on the amount of nitrogen (in grams per minute) obtained under process conditions for a given degree of ammonia dissociation and a given inlet atmosphere flow rate. The concept of availability is defined in [4] and described by the formula:

(1) $$m_{N_{2}}\left(t\right)=\frac{1}{6}\cdot \frac{\alpha \left(t\right)}{2-\alpha \left(t\right)}\cdot F_{w}\left(t\right)$$

where α—degree of ammonia dissociation and F_w_—inlet atmosphere flow rate.

The nitriding potential, degree of dissociation, and nitrogen availability can be used to control the nitrogen flux reaching the nitrided steel surface.

In both single-component and two-component atmospheres, the Np value can be modified by changing the flow rate, which affects the availability of nitrogen. In addition, the Np value can be modified—depending on the type of process—either by increasing the dilution ratio of NH with dissociated NH_3_ or by diluting NH_3_ with N_2_. In the first of these variants, regardless of the degree of dilution, the same value of Np will correspond to the same value of available nitrogen [14]. In the second variant, the availability of nitrogen will change, while the value of the potential will not change [15,16,17].

The aim of this research, the results of which are discussed in this paper, was to compare two-component inlet atmospheres diluted with either hydrogen (NH_3_/H_2_) or nitrogen (NH_3_/N_2_).

Taking advantage of the features of inlet atmospheres diluted with nitrogen and hydrogen, four two-stage processes were designed and carried out. A common parameter of the processes carried out was the same value of nitrogen availability in each process stage.

In the NH_3_/H_2_ inlet atmosphere, the availability was adjusted by changing the degree of dilution of the inlet atmosphere with hydrogen, while keeping the flow rate of the inlet atmosphere constant. In the NH_3_/N_2_ inlet atmosphere, the nitrogen availability was adjusted by changing the degree of dilution of the inlet atmosphere with nitrogen, also while keeping the flow rate of the inlet atmosphere constant.

The research results will increase knowledge regarding the possibility of using nitrogen and hydrogen to control the phase composition and growth kinetics of iron nitride layers formed on AISI 1085 unalloyed and AISI 52100 alloy steel.

## 2. Materials and Methods

The materials used for the tests were 3 mm diameter balls made of AISI 1085 and AISI 52100 steel. Basic information on their chemical compositions is included in Table 1.

In terms of technological research, 6 nitriding processes were carried out as follows: two single-stage processes in ammonia (1, 4) and four two-stage processes (2, 3, 5, 6). The one-stage processes (1 and 4) were carried out only in an NH_3_ atmosphere. The first stage of the two-stage processes (2, 3, 5, 6) was also carried out in an NH_3_ atmosphere, while the second stage of processes 2 and 5 was carried out in an NH_3_/H_2_ atmosphere and of processes 3 and 6 in an NH_3_/N_2_ atmosphere. In each condition, 20 samples were nitrided. The parameters of the above processes are compiled in Table 2. The parameters were based on previously conducted research [6,14,18,19] and fill the existing gap.

**Figure 1:**
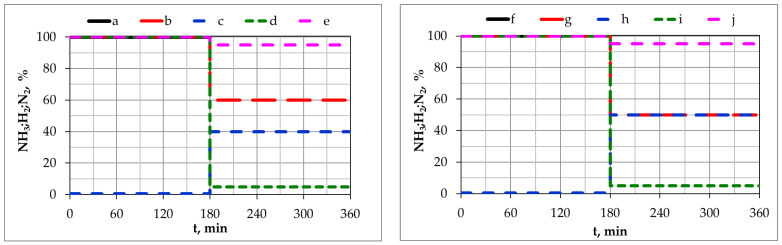
Change in NH_3_, H_2_, and N_2_ contents in the inlet atmosphere in process 1: NH_3_ (a), process 2: NH_3_ (b) and H_2_ (c), process 3: NH_3_ (d) and N_2_ (e), process 4: NH_3_ (f), process 5: NH_3_ (g) and H_2_ (h), and process 6: NH_3_ (i) and N_2_ (j).

Figure 1 shows the change in NH_3_, H_2_, and N_2_ contents in the inlet atmosphere in the individual processes.

**Figure 2:**
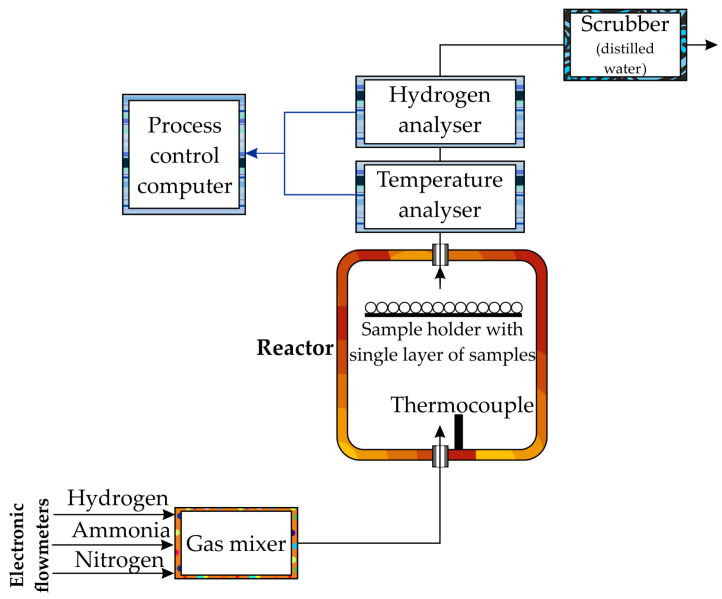
General diagram of the functioning of a thermobalance.

The processes were carried out using a thermobalance (a chemical reactor with a precise thermogravimetric measurement of 50 µg). The apparatus is computer-controlled, and the sample undergoing the process is placed in a crucible on the pan of the measuring system in an electrically heated cylindrical chamber (Figure 2). A more extensive characterisation of the apparatus is available in another article [19].

During nitriding, the reactor measuring system recorded the process temperature, NH_3_, H_2_, and N_2_ flow rates, and H_2_ content in the outlet atmosphere. 

In the nitriding processes in the NH_3_ and NH_3_/H_2_ atmospheres, the nitrogen potential value (Np) was calculated from the relationship:

(2) $$N_{p}=\frac{1-\frac{4}{3}\cdot Q}{Q^{1.5}}$$

while in the case of nitriding in the NH_3_/N_2_ atmosphere, the following relationship was used:

(3) $$N_{p}=\frac{1-b-\frac{2}{3}\cdot Q\left(2-b\right)}{Q^{1.5}}$$

where Q—hydrogen content in the outlet atmosphere (volume fraction) and b—nitrogen content in the inlet atmosphere NH_3_/N_2_.

The selection of nitriding process parameters in the range of nitrogen potential values was established using the Lehrer phase equilibrium diagram. In single-stage processes and the first stage of the two-stage processes, the nitrogen potential value was maintained from the phase stability area ε. In the second stage of the two-stage processes in the NH_3_/H_2_ atmosphere, the nitrogen potential value was maintained in the phase stability area γ’.

**Figure 3:**
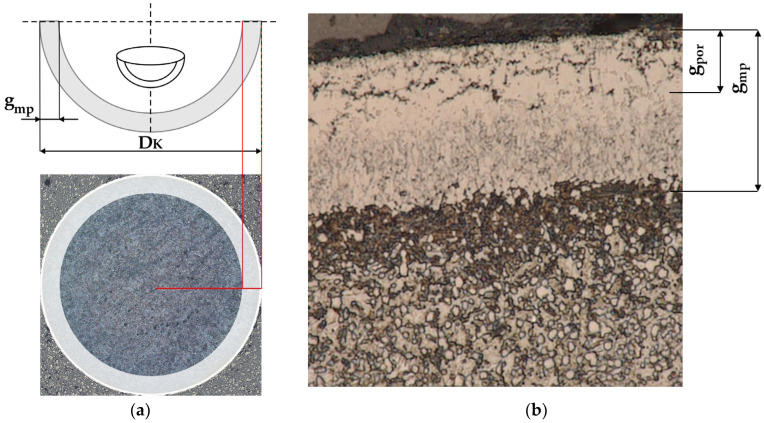
(**a**) Metallographic cross-section of a ball sample at its diameter and (**b**) the microstructure of AISI 52100 steel with an iron nitride layer.

In order to measure the actual thickness of the obtained nitride layers, the balls were subjected to a grinding process to their diameter. The detailed course of this process along with an example of the microstructure is shown in Figure 3. The image shows the total thickness of the iron nitride layer (g_mp_) with an indication of the porous zone (g_por_) (Figure 3b).

The processes carried out led to the achievement of various thicknesses of the iron nitride layer (white layer), various thicknesses of the porous in the iron nitride, and various white layer hardness values. The geometries of the samples after nitriding are presented in Table 3. 

An X-ray diffractometer was used to determine the phase composition of the obtained iron nitride layers. The measurements used symmetrical geometry and KαCo radiation.

The morphology and distribution of nitrides in the cross-section of the materials were studied using the Hitachi S-3400N Scanning Electron Microscope (Tokio, Japan). The accelerating voltage during the experiments was 15 keV. The depth of penetration of electrons depends, among other things, on their energy. The depth of penetration of the beam into materials at 15 KV is approximately 1 µm. The chemical composition was studied using a scanning electron microscope equipped with an EDS X-ray analyser from AMETEK GmbH (Berwyn, PA, USA). Qualitative and quantitative analyses were carried out using an EDS Element X-ray spectrometer and analysed with APEX™ software using the eZAF correction method. The EDS detector for the study of chemical composition allows the detection of minimal beryllium (Be) elements and has a minimum resolution of 129 eV for the Mn (Kα) line. The window of the EDS detector is made of silicon nitride—Si_3_N_4_ with a thickness of less than 100 nm—and thus has an increased sensitivity for the detection of light elements.

## 3. Results

The aim of this study was to assess the effect of diluting the ammonia inlet atmosphere with hydrogen or nitrogen on the mass change in nitrided samples and the influence of these parameters on the thickness and structure of the subsurface layer of iron nitrides. The purpose of recording the mass change in the samples online during nitriding was to investigate whether and to what extent the dilution of the ammonia inlet atmosphere with hydrogen or nitrogen, during process stage II, would affect the course of the mass change in the nitrided samples.

Nitriding in the NH_3_ atmosphere was carried out in a single-stage process at 590 °C for 3 h, maintaining the nitriding potential in the ε phase stability region. Studies in the NH_3_/H_2_ and NH_3_/N_2_ atmospheres were carried out in two-stage processes. The parameters in the first stage were the same as in the single-stage process, while the second stage was carried out in the NH_3_/H_2_ atmosphere at a nitriding potential from the γ’ phase stability region, and in the NH_3_/N_2_ atmosphere, as in the single-stage process, the value of the nitriding potential was maintained in the ε phase stability region.

### 3.1. Nitriding of AISI 1085 Steel

**Figure 4:**
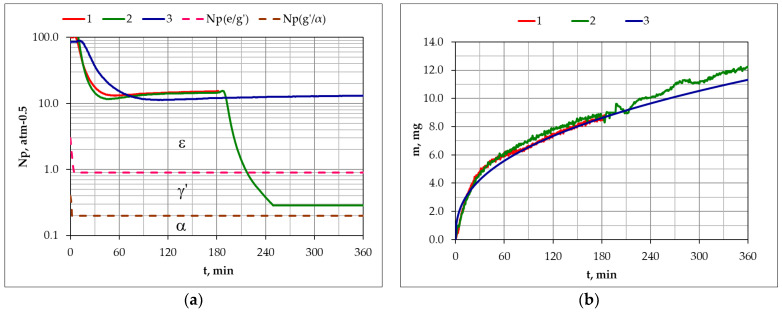
Change in the (**a**) values of nitriding potential as a function of time in the single-stage process—process 1, in the two-stage process in the NH_3_/H_2_ atmosphere—process 2, in the NH_3_/N_2_ atmosphere—process 3, Np(ε/γ’), Np(γ’/α), respectively, the functions of boundaries (ε/γ’) and (γ’/α) according to Lehrer’s equilibrium system, and the (**b**) masses of nitrided AISI 1085 steel samples in the single-stage process—process 1, in the two-stage process in the NH_3_/H_2_ atmosphere—process 2, and in the NH_3_/N_2_ atmosphere—process 3.

Figure 4 shows the course of changes in nitriding potential as a function of process time in the single-stage and two-stage processes (Figure 4a), as well as the course of the changes in the mass of the nitrided samples in the aforementioned processes. The values of the nitriding potential (Figure 4a) in the single-stage process (1), in the first stage of the process (2) and during the whole process (3), corresponded to the ε phase stability. Dilution of the inlet atmosphere with hydrogen in the second stage of the process (2) reduced the nitriding potential to a value corresponding to the γ’ phase stability. Reducing the potential value in the second stage of the process did not reduce the mass growth kinetics of the samples. 

Dilution of the NH_3_ atmosphere with nitrogen in the second stage of process 3 did not reduce the nitriding potential value (Figure 4a). The consequence of diluting the inlet atmosphere with nitrogen was a reduction in nitrogen availability. The reduction in nitrogen availability did not, similarly to the reduction in the nitriding potential value, reduce the mass growth kinetics of the samples. The mass increase in the samples, in both single-stage and two-stage processes, can be described by a power function:

m(t) = 0.123 ∙ t^0.38^ (4)

**Figure 5:**
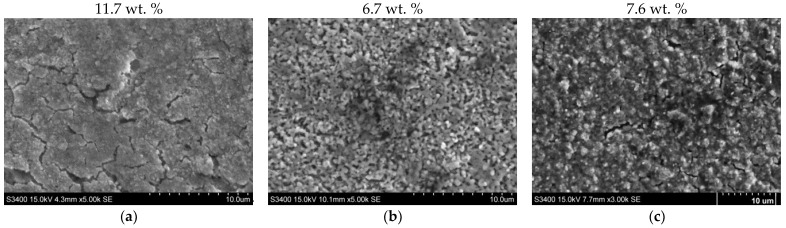
Surface appearance of AISI 1085 steel: (**a**) nitrided in the single-stage process for 3 h in the NH_3_ atmosphere—process 1; (**b**) nitrided in the two-stage process, 1st st., 3 h, NH_3_ atmosphere: 2nd st., 3 h, NH_3_/H_2_ atmosphere—process 2; and (**c**) 2nd st., 3 h, NH_3_/N_2_ atmosphere—process 3. The photos show the nitrogen concentration in the subsurface zone of the iron nitride layer in percentage by weight.

Figure 5 shows the surface appearance of a sample of AISI 1085 steel that was nitrided in the single-stage process (Figure 5a), the two-stage process in the NH_3_/H_2_ atmosphere in the second stage (Figure 5b), and the two-stage process in the NH_3_/N_2_ atmosphere in the second stage (Figure 5c). The nitrogen content on the surface of the nitrided layer (Figure 5a) indicates that the iron nitride layer of the nitrided sample in the single-stage process has a monophase ε-zone, while after nitriding in the two-stage processes, the subsurface zone has a mixture of ε + γ’ phases. The surface of sample 1 is porous. Porosity develops during layer growth because of the recombination of atomic nitrogen into molecular nitrogen, and then, during cooling, cracks may become visible on the surface of the layer. The surface morphology of iron nitrides formed by two-stage processes (Figure 5b,c) differs from that of iron nitrides formed by single-stage processes (Figure 5a). All changes in surface morphology that formed in the second stage of the process are the result of partial denitrification of the iron nitride layer in the second stage of the process. As a result of these processes, so-called secondary microporosity is formed on the surface. In process 2, as a result of hydrogen involvement in the denitrification of the iron nitride layer, the surface porosity is significantly higher than that of the surface after process 3.

**Figure 6:**
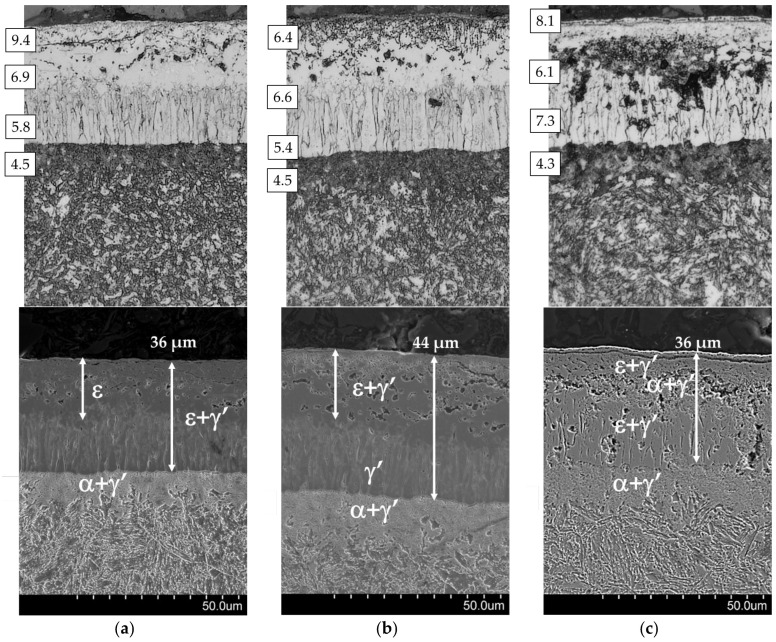
Microstructure of AISI 1085 steel: (**a**) nitrided in the single-stage process for 3 h in the NH_3_ atmosphere—process 1; (**b**) nitrided in the two-stage process, 1st st., 3 h, NH_3_ atmosphere: 2nd st., 3 h, NH_3_/H_2_ atmosphere—process 2; and (**c**) 2nd st., 3 h, NH_3_/N2 atmosphere—process 3. The photos show the nitrogen concentration in the subsurface zone of the iron nitride layer in percentage by weight.

Figure 6 shows the microstructures of AISI 1085 steel, which was nitrided in the single-stage process (Figure 6a), the two-stage process in the NH_3_/H_2_ atmosphere in the second stage of the process (Figure 6b), and the two-stage process in the NH_3_/N_2_ atmosphere in the second stage of the process (Figure 6c). The processes produce layers with thicknesses of 36, 44, and 36 µm, respectively. Depending on the process variant, there are differences in nitrogen concentrations in the different zones of the iron nitride layer. 

In the iron nitride layer formed in the single-stage process (Figure 6a), a porous ε zone with a thickness of 15–20 µm can be distinguished; the porosity of the zone decreases towards the boundary of the ε + γ’ zone with a palisade structure perpendicular to the substrate.

In the second stage (Figure 6b) of the two-stage process in the NH_3_/H_2_ atmosphere, it is possible to distinguish a 20–26 µm thick ε + γ’ zone, which formed in the second stage of the process. As a result of the partial transformation of the ε phase into the γ’ (ε→ε + γ’ + N↑) phase, the nitrogen formed during the transformation, after recombining into N_2_, passes into the atmosphere, causing an increase in porosity compared with the ε zone in Figure 6a. Beneath the ε + γ’ zone, a palisade-like γ’ zone formed as a result of the transformation $\varepsilon +\gamma^{\prime }\to \gamma_{\varepsilon }^{\prime }+\gamma^{\prime }+N↑$. The transformation resulted in a slight increase in the porosity of this zone.

In the second stage (Figure 6c) of the two-stage process in the NH_3_/N_2_ atmosphere, a single ε + γ’ zone formed, up to 10 µm thick. Beneath this zone, a second zone of eutectoid α + γ’ (darkly etched so-called braunite with a thickness of 5–25 µm) formed. Beneath this zone, a third zone of mixed iron nitrides ε + γ’ was present. 

It should be emphasised that a braunite (α + γ’) zone formed on all AISI 1085 steel samples directly under the iron nitride layer. This is probably due to exceeding the eutectoid temperature, i.e., 590 °C, from the Fe-N equilibrium system.

**Figure 7:**
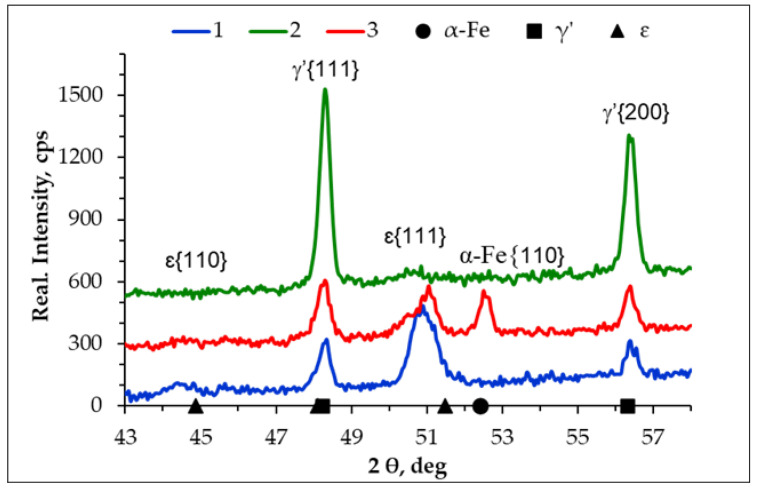
Diffractograms from the surface of AISI 1085 steel samples: nitrided in the single-stage process for 3 h in the NH_3_ atmosphere (1), nitrided in the two-stage process, 1st st., 3 h, NH_3_ atmosphere: 2nd st., 3 h, NH_3_/H_2_ atmosphere (2), and 2nd st., 3 h, NH_3_/N_2_ atmosphere (3).

Figure 7 shows diffractograms from the surface of the AISI 1085 steel samples nitrided in the single-stage process in the NH_3_ atmosphere (1), the two-stage process in the NH_3_/H_2_ atmosphere in the second stage (2), and the two-stage process in the NH_3_/N_2_ atmosphere (3). On samples 1 and 3, the ε and γ’ phases were identified; on sample 2, only the γ’ phase was identified. However, there was also a peak from iron on sample 3, which was not a peak from the substrate, as the penetration range of the CoKα lamp radiation is 17 µm, as evidenced by the absence of peaks from iron on the diffractograms of samples 1 and 2. The thickness of the iron nitride layers obtained on these samples was greater than 17 µm. Instead, the presence of peaks from iron in the iron nitride layer indicated the presence of braunite, i.e., the α + γ’ eutectoid. The results of the X-ray phase analysis correlated well with those obtained using an EDS X-ray analyser. In the iron nitride layer formed in the single-stage and two-stage processes, the percentages of the ε and γ’ phases were 78 and 22 vol%, respectively. In the iron nitride layer formed in the two-stage process, in which the second stage proceeded in an NH_3_/H_2_ atmosphere as a result of the reaction of hydrogen with the ε($\varepsilon \overset{H_{2}}{\to }\;\gamma \prime +N↑\;$) phase, the volume fraction of the γ’ phase in the iron nitride layer increases. Consequently, after completion of the second stage of the process, in the iron nitride layer, the volume fraction of the ε phase did not exceed a few per cent.

### 3.2. Nitriding of AISI 52100 Steel

**Figure 8:**
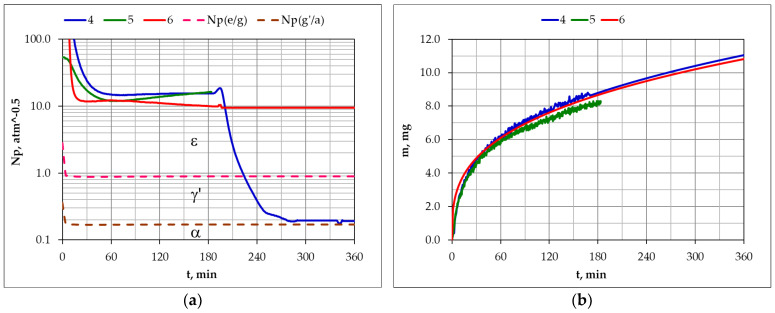
Change in the (**a**) nitriding potential as a function of nitriding time in the single-stage process—process 4, in the two-stage process in the NH_3_/H_2_ atmosphere—process 5, and in the NH_3_/N_2_ atmosphere—process 6. (**b**) Change in the mass of nitrided AISI 52100 steel samples in the single-stage process—process 4, in the two-stage process in the NH_3_/H_2_ atmosphere—process 2, and in the NH_3_/N_2_ atmosphere—process 3. Np(ε/γ’) and Np(γ’/α), respectively, are the functions of boundaries (ε/γ’) and (γ’/α) according to Lehrer’s equilibrium system.

Figure 8 shows the course of changes in nitriding potential as a function of process time in the single-stage and two-stage processes (Figure 8a) and the course of changes in the mass of the nitrided samples in these processes (Figure 8b). The values of the nitriding potential (Figure 8a) differ slightly in the single-stage process (4), in the first stage of the process (5), and during the whole process (6), and their values corresponded to the ε phase stability at the process temperature. 

Dilution of the inlet atmosphere with hydrogen in the second stage of the process (4) resulted in a reduction in the nitriding potential to a value corresponding to the γ’ phase stability. Reducing the potential value in the second stage of the process did not reduce the mass growth kinetics of the samples. 

Dilution of the NH_3_ atmosphere with nitrogen in the second stage of the process (6) did not reduce the nitriding potential value (Figure 8a). The consequence of diluting the inlet atmosphere with nitrogen is a reduction in nitrogen availability. The reduction in nitrogen availability also did not, similarly to the reduction in the nitriding potential value, reduce the mass growth kinetics of the samples. The mass increase in the samples, in both single-stage and two-stage processes, can be described by a power function:

m(t) = 0.387 ∙ t^0.336^ (5)

**Figure 9:**
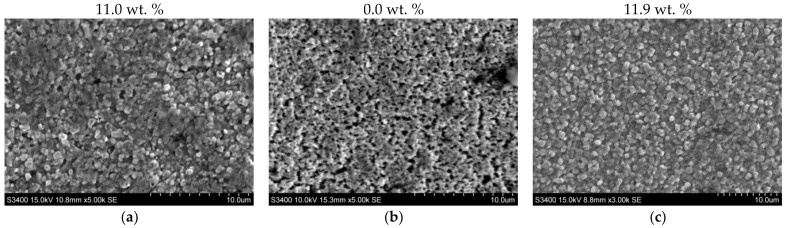
Surface appearance of AISI 52100 steel (**a**) nitrided in the single-stage process for 3 h in the NH_3_ atmosphere—process 4; (**b**) nitrided in the two-stage process, 1st st., 3 h, NH_3_ atmosphere: 2nd st., 3 h, NH_3_/H_2_ atmosphere—process 5, and (**c**) 2nd st., 3 h, NH_3_/N_2_ atmosphere—process 6. The photos show the nitrogen concentration in the subsurface zone of the iron nitride layer in percentage by weight.

Figure 9 shows the surface appearance of the AISI 52100 steel sample, which was nitrided in the single-stage process (Figure 9a), the two-stage process in the NH_3_/H_2_ atmosphere in the second stage of the process (Figure 9b), and the two-stage process in the NH_3_/N_2_ atmosphere (Figure 9c) in the second stage of the process. The nitrogen content on the surface of the iron nitride layer after the single-stage process in ammonia (Figure 9a), and on the surface of the iron nitride layer after the two-stage process in the NH_3_/N_2_ atmosphere in the second stage (Figure 9c), indicates that there is a monophase ε-zone in the subsurface zone of the iron nitride layer. No nitrogen was found on the surface of the sample after nitriding by the two-stage process in the NH_3_/H_2_ atmosphere in the second stage, but oxygen 4.5 wt.% and iron above 90 wt.% were found.

The surface morphologies of the iron nitride layers formed in the single-stage process (Figure 9a) and the two-stage process in the NH_3_/N_2_ atmosphere in the second stage (Figure 9c) were similar. The surfaces of the samples were covered with uniformly sized ε-Fe_3_N nitride crystallites. On the surface of the steel after the two-stage process in the NH_3_/H_2_ atmosphere in the second stage, microporosity was visible, formed by nitrogen, which was the product of the total reduction reaction of ε-Fe_3_N nitride with hydrogen.

**Figure 10:**
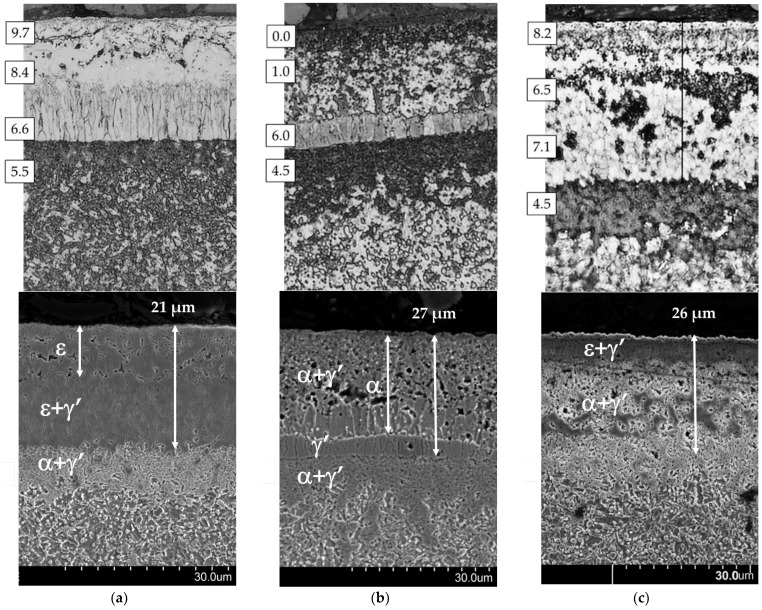
Microstructure of AISI 52100 steel (**a**) nitrided in the single-stage process for 3 h in the NH_3_ atmosphere—process 4; (**b**) nitrided in the two-stage process, 1st st., 3 h, NH_3_ atmosphere: 2nd st., 3 h, NH_3_/H_2_ atmosphere—process 5, and (**c**) 2nd st., 3 h, NH_3_/N_2_ atmosphere—process 6. The photos show the nitrogen concentration in the subsurface zone of the iron nitride layer in percentage by weight.

Figure 10 shows the microstructures of AISI 52100 steel, which was nitrided in the single-stage process (Figure 10a), the two-stage process in the NH_3_/H_2_ atmosphere in the second stage (Figure 10b), and the two-stage process in the NH_3_/N_2_ atmosphere in the second stage of the process (Figure 10c). The layers obtained were 21, 27, and 26 µm thick, respectively. The images show the zones of measurement of nitrogen concentration in the iron nitride layer. Depending on the process variant, there were significant differences in nitrogen concentrations in the different zones of the iron nitride layer.

In the iron nitride layer formed in the single-stage process (Figure 10a), we distinguished a porous ε zone near the surface of the layer with a thickness of 10–12 µm and an ε + γ’ zone. In the second stage (Figure 10b) of the two-stage process in the NH_3_/H_2_ atmosphere, a 21–24 µm thick α_N_ zone formed. As can be seen from the nitriding potential values, when the first stage of the nitriding process was completed, the dominant phase in the iron nitride layer that formed was ε. Lowering the nitriding potential to the γ’/α phase interface initiated the reduction of the ε phase to γ’ ($\varepsilon \overset{H_{2}}{\to }\;\gamma \;\prime +N↑\;$), followed by the reduction of the γ’ phase to α_N_. In the presence of hydrogen, this phase transformation—$\;\gamma \;\prime \overset{H_{2}}{\to }\alpha_{N}+N↑$—occurs through a chemical reaction. The nitrogen formed as a result of the reduction, after recombining to molecular form, enters the atmosphere, causing an increase in porosity in this zone of the iron nitride layer. In the area of the α_N_ zone, there are areas where α + γ’ eutectoid (braunite—strongly etched in the α_N_ zone) formed during the second stage of the process. The non-denitrified zone γ’, 3–6 µm thick with a palisade structure perpendicular to the substrate, remained a subzone of α_N_.

In the second stage of the two-stage process in the NH_3_/N_2_ atmosphere, an ε + γ’ zone with a thickness of approximately 6 µm formed, and directly below it, there was a braunite zone (α + γ’ eutectoid) and α areas.

As in the case of AISI 1085 steel, a zone of the α + γ’ eutectoid formed under the iron nitride layer in all AISI 52100 steel samples.

**Figure 11:**
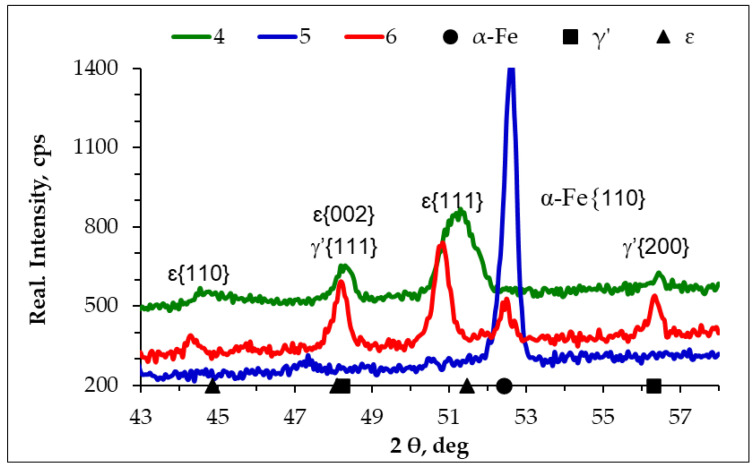
Diffractograms from the surface of AISI 52100 steel samples nitrided in the single-stage process for 3 h in the NH_3_ atmosphere (4), nitrided in the two-stage process, 1st st., 3 h, NH_3_ atmosphere: 2nd st., 3 h, NH_3_/H_2_ atmosphere (5), and 2nd st., 3 h, NH_3_/N_2_ atmosphere (6).

Figure 11 shows diffractograms from the surface of the AISI 52100 steel samples nitrided in the single-stage process in the NH_3_ atmosphere (4), the two-stage process in the NH_3_/H_2_ atmosphere in the second stage (5), and the two-stage process in the NH_3_/N_2_ atmosphere (6). The ε and γ’ phases were identified on samples 4 and 6. 

In the iron nitride layer formed in the two-stage process in the NH_3_/H_2_ atmosphere, only one peak from iron (α-Fe) appeared on the diffractogram in the second stage. The absence of a peak from γ’ on the diffractogram can be explained by the limited depth of penetration of CoKα X-rays—the maximum penetration is 17 µm—while the γ’ zone is located at a distance of 21 µm from the surface. The recorded peaks from iron on the diffractograms from the surfaces of samples 5 and 6, which do not originate from the substrate, confirm the presence of the α + γ’ eutectoid in the above samples.

## 4. Discussion

In summary, it must be stated that for the nitriding of AISI 1085 non-alloy steel in an ammonia (NH_3_) atmosphere, both in the single-stage process (process 1) and in the first stage of process 2, as well as during the entire second stage of process 3, nitriding potential values favourable for the formation of the ε phase were obtained. On the other hand, dilution of the inlet atmosphere with hydrogen (NH_3_/H_2_), in the second stage of process 2 resulted in a reduction in the nitriding potential to a value corresponding to the γ’ phase stability. However, it should be emphasised that such a significant reduction in the nitriding potential value did not reduce the mass growth kinetics of the nitrided samples. In contrast, diluting the atmosphere with nitrogen (NH_3_/N_2_), resulted in a reduction in nitrogen availability without changing the nitriding potential value, which did not, however, slow down the mass growth kinetics of the samples, as in the case of diluting the ammonia atmosphere with hydrogen.

When analysing the above nitriding processes in the context of the structures obtained, it was noticeable that single-stage nitriding results in the formation of a monophasic porous ε phase in the surface layer. In contrast, the dilution of ammonia with hydrogen or nitrogen resulted in a mixture of ε + γ’ phases in the subsurface zone. The presence of a porous and monophase ε phase on the surface of the nitrided element requires the use of an additional impregnation procedure with corrosion inhibitors or the nitrocarburizing process after the nitriding process. This increases the costs of adapting nitrided elements to operation in conditions of external exposure to the surrounding aggressive environment.

In contrast, the nitriding of AISI 52100 alloy steel showed many similarities but also fundamental differences compared with the nitriding of ASIS 1085 non-alloy steel. As in the case of non-alloy steel, during the nitriding of AISI 52100 alloy steel in the single-stage process (4), in the first stage of the process (5) and during the entire two-stage process (6), the potential values were responsible for the formation of a porous ε phase layer in the surface zone. 

Dilution of the inlet atmosphere with hydrogen as well as with nitrogen in the second stage of the process also—as in the case of non-alloy steel—reduced the nitriding potential to a value corresponding to the γ’ phase stability, which, however, did not reduce the mass growth kinetics of the samples. In contrast, a significant difference was found for the two-stage process (5) in the NH_3_/H_2_ atmosphere, in which no nitrogen was found on the surface in the second stage, but oxygen 4.5 wt.% and iron above 90 wt.% were found. This could indicate that the iron nitride layer in this two-stage process underwent complete decomposition, which was not found in ASIS 1085 steel. Dissorption of nitrogen from the nitrided surface is, of course, an unfavourable phenomenon; therefore, two-stage nitriding with the parameters adopted in the process (5) cannot be used in industrial conditions because it leads to undesirable decomposition of the nitrided layer. Too high a share of hydrogen in the gas mixture used is therefore unfavourable.

An increase in the mass of the samples was observed in all nitriding processes carried out, but there were clear differences in the thickness of the iron nitride layers obtained. These differences are due to the fact that in AISI 1085 non-alloy steel, because of the low solubility of nitrogen in ferrite, a greater mass of absorbed nitrogen accumulates in the iron nitride layers. On the other hand, in AISI 52100 alloy steel, the presence of chromium results in increased nitrogen absorption by the substrate because of the formation of chromium carbonitrides. Thus, the observed phenomenon—in relation to steel alloys containing alloying elements that form carbonitrides or nitrides of these elements during nitriding—promotes nitrogen adsorption into the substrate. In addition, analysis of the distribution of elements in the surface layer can be a very helpful method for determining the phase structure of the obtained nitrided layers. Knowing that transition metal nitrides have similar properties and structural structure to carbides, and based on the knowledge of the distribution of the chemical composition and the probable analogy to the classification of carbides according to Goldschmidt’s criteria, we can determine the probable phase structure of the obtained layers. Analogously to the classification of carbides, it should be assumed that the transition metal elements located further to the left of the periodic table (lower group) are more nitriding compared with those lying on the right side.

## 5. Conclusions

The research and analysis of the findings lead to the following conclusions and observations:

1. The chemical composition of the nitrided substrate for the adopted nitriding process parameters does not affect the kinetics of the growth of the mass of nitrided samples as a function of the nitriding time.
2. The alloying additives present in the steels studied significantly affect the nitrogen distribution between the resulting iron nitride layer and the diffusion zone in the nitrided substrate.
3. Dilution of the ammonia inlet atmosphere with hydrogen initiates phase transformations of the iron nitrides through a chemical reaction.
4. Dilution of the ammonia atmosphere with nitrogen initiates phase transformations of the iron nitrides within their thermodynamic stability at a given nitriding process temperature.

The results of this research can be used in practice for the selection of nitriding atmospheres used in gas nitriding processes aimed at obtaining iron nitride layers with the required phase composition. The thickness and phase composition of iron nitride layers are of significant importance in nitriding processes, including anticorrosion nitriding. Control of the phase composition of iron nitrides can be used to control the magnetic properties. The concentration of nitrogen in the ε-phase significantly affects its magnetic properties. Diluting ammonia with nitrogen makes it possible to reduce the cost of the nitriding process. In addition, even partial replacement of ammonia with nitrogen is of great importance from an environmental point of view.

## Figures and Tables

**Table 1:** The chemical composition of the steel used in the tests.

Steel Grade	D ^1^ (mm)	Element Content in wt.%						
		C	Si	Mn	S	P	Cr	Fe
AISI 52100	3.0	1.0	0.3	0.45	0.014	0.02	1.5	rest
AISI 1085	3.0	0.84	0.3	0.80	0.040	0.03	-	rest

^1^ ball diameter.

**Table 2:** Nitriding parameters.

Sample No.	T (°C)	T (min)	Inlet Atmosphere			Steel Grade
			HN_3_, %	N_2_, %	H_2_, %	
1	590	180	100	0	0	AISI 1085
2	590	180	100	0	0	AISI 1085
	590	180	60	0	40	
3	590	180	100	0	0	AISI 1085
	590	180	5	95	0	
4	590	180	100	0	0	AISI 52100
5	590	180	100	0	0	AISI 52100
	590	180	50	0	50	
6	590	180	100	0	0	AISI 52100
	590	180	5	95	0	

**Table 3:** Geometries of the samples after nitriding.

Sample No.	WL ^1^ (µm)	WL por ^2^ (µm)	Phase Composition of WL	HWL ^3^ HV0.1	Δm mg	Steel Grade
1	36	15–20	ε + γ’	550	9	AISI 1085
2	41	20–27	γ’	738	12	AISI 1085
3	36	36	ε + γ’, α + γ’	-	10.7	AISI 1085
4	21	10	ε + γ’	589	8	AISI 52100
5	27	21–24	α-Fe (γ’)	591	8.8	AISI 52100
6	28	24–27	ε + γ’, α + γ’	-	9.0	AISI 52100

^1^ Thickness of the iron nitride layer (white layer). ^2^ Thickness of the porous in the iron nitride layer. ^3^ White layer hardness.

## Data Availability

The data can be accessed from Czestochowa University of Technology.
